# Congenital Rubella Syndrome in Fiji, 1995–2010

**DOI:** 10.1155/2013/956234

**Published:** 2013-02-03

**Authors:** Sheetalpreet Singh, Frances Bingwor, Katherine Tayler-Smith, Marcel Manzi, Guy B. Marks

**Affiliations:** ^1^Health Information Unit, Division of Health Information, Research and Analysis, Ministry of Health, Government Buildings, P.O. Box 2223, Suva, Fiji; ^2^Family Health Programme, Public Health Division, Ministry of Health, Government Buildings, P.O. Box 2223, Suva, Fiji; ^3^Operational Research, Medical Department, Operational Centre of Brussels, Medecins Sans Frontieres Luxembourg (MSFL), 1617 Luxembourg, Luxembourg; ^4^Woolcock Institute of Medical Research, Missenden Road, P.O. Box M77, Sydney, NSW 2050, Australia; ^5^Department of Respiratory Medicine, Liverpool Hospital, Locked Bag 7103, Liverpool, NSW BC 1871, Australia

## Abstract

*Setting*. A nationwide study in Fiji. *Objective*. To describe the incidence of congenital rubella syndrome (CRS) and its relationship to the incidence of notified cases of rubella in Fiji from 1995 to 2010. *Design*. Descriptive, retrospective review of all recorded congenital abnormalities associated with live births in Fiji over 16 years. *Results*. There were 294 infants who met the criteria for CRS. Of these, 95% were classified as “suspected” cases, 5% were “clinically confirmed,” and none were “laboratory confirmed cases”. There was a significant linear increase over the study period in the incidence of CRS (odds ratio 1.045 per year, 95% CI 1.019 to 1.071, *P* ≤ 0.001). There was no significant association between the incidence of CRS and the reported incidence of rubella (*P* = 0.3). *Conclusion*. There is a rising trend in reports of suspected CRS cases in Fiji. This highlights the need to strengthen surveillance for CRS through improvements in clinical and laboratory diagnosis to confirm or exclude suspected cases. It is also important to ensure high coverage of rubella vaccination in Fiji.

## 1. Introduction

Whilst rubella is usually a mild disease in adults and children, maternal infection with rubella, especially early in pregnancy, can cause severe defects in the developing foetus, resulting in congenital rubella syndrome (CRS). The constellation of anomalies of CRS includes ophthalmic, auditory, cardiac, and craniofacial defects [1].

CRS is common in developing countries, affecting about 110,000 infants annually in these countries [2]. In 2009 there were 121,344 cases of rubella reported from 167 WHO member countries [2]. In addition, 165 cases of CRS were reported to WHO by 123 member countries in the same year [2]. In the Western Pacific Region, the number of rubella cases increased 12-fold from 5475 in 2000 to 73077 in 2009. With rubella a growing problem in the Western Pacific Region, there are concerns that CRS may also be on the rise in this region.

The relationship between the incidence of rubella and the incidence of CRS has not been clearly shown, although some studies from resource poor settings, such as Romania, have shown clusters of children with CRS after rubella outbreaks [2]. In Fiji, the incidence of rubella has ranged between 1 and 30 cases per 100,000 population with outbreaks noted in 1995, 2002, 2006, and 2011 [3]. The most recent rubella outbreak, in July 2011, has highlighted the need to carry out surveillance of CRS in Fiji. Vaccination against rubella was introduced in 1975 for females (at 12 years of age), and in 2004 this was extended to include both males and females at primary school entry [4]. However, a lack of information on the incidence of CRS in Fiji limits our ability to assess the effectiveness of these vaccination campaigns. 

To address this information gap, this study seeks to (a) report on the incidence of CRS in Fiji and the relationship between the incidence of CRS and the incidence of notified cases of rubella since 1995 and (b) document how CRS cases are classified (suspected (possible), clinically confirmed (probable), or laboratory confirmed (definite)) over the period 1995–2010. 

## 2. Methods

### 2.1. Design

This was a descriptive study involving a retrospective review of all recorded congenital abnormalities associated with live births in Fiji over a 16-year period (1995–2010).

### 2.2. Setting

Fiji is an island nation located in the South-West Pacific with a population of approximately 837,271 [5]. It consists of approximately 332 islands covering a total land area of about 18,333 sqkm [5]. The main sources of revenue are from tourism and primary industry [5]. 

The Ministry of Health (MoH) in Fiji provides decentralized health services through a three tier structure of primary, secondary, and tertiary care. Fiji's health system comprises three divisional hospitals, 17 subdivisional hospitals, 78 health centres, and 103 nursing stations [5]. There is a hierarchical referral mechanism from nursing stations to health centres to subdivisional and divisional hospitals. Infants born with congenital defects requiring intervention are likely to be referred to the paediatric departments in the three divisional hospitals—Colonial War Memorial (CWM) Hospital, Lautoka Hospital, and Labasa Hospital. Infants with newly diagnosed congenital anomalies are routinely admitted to intensive care wards at these hospitals. 

### 2.3. Sample

The study population included all live births in Fiji with a congenital anomaly registered between January 1, 1995, and December 31, 2010.

### 2.4. Data Collection

#### 2.4.1. Data Sources

Between November 2011 and April 2012, data were sourced from the three divisional hospitals. All newborn infants with congenital anomalies were identified from the Congenital Anomalies registers when these registers were available, and when not available, from a review of the Neonatal Intensive Care Unit registers. Data on the number of live births and the annual population numbers were obtained from MoH Consolidated Monthly Reports (CMR) for the period 1995–2008 and from the MoH Public Health Information System (PHIS) for the period 2009-2010. Data on the annual number of reported cases of rubella were obtained from the National Notifiable Disease Surveillance System Reports for the period 1995–2010. The CMR and PHIS records were used to allow calculation of the incidence rates of CRS and rubella. 

#### 2.4.2. Variables

For each recorded case of CRS the following data were collected: hospital, date registered, date of birth, sex, ethnicity, and description of congenital defects. The presence or absence of specific congenital defects was recorded, as specified in the WHO diagnostic classification for CRS [1] and cases were classified as “suspected,” “clinically confirmed,” or “laboratory confirmed” (see Box 1). 

#### 2.4.3. Data Management and Validation

Data was directly entered into an Excel spreadsheet. Patient notes were available for some cases of CRS diagnosed from 2002 onwards. Where these notes were available, they were reviewed to verify the diagnosis that had been recorded in the Congenital Anomalies book or the Neonatal Intensive Care Unit register. 

### 2.5. Analysis and Statistics

The validated dataset was analysed in Microsoft Excel. Pivot tables were used to tabulate CRS and rubella cases by year of registration. The incidence of CRS was expressed per 1000 live births, and the incidence of rubella was expressed as cases per 100,000 population for each year. 

The association between the incidence of rubella and the incidence of CRS was estimated by linear regression using Epi-Info, version 3.5.1.

## 3. Results

The Congenital Anomalies book was not available for Lautoka Hospital and for Labasa Hospital before 2010. Data was also missing for CWM Hospital for 1998 and June–December for 2000. 

Based on the available data, a total of 977 babies with congenital anomalies were recorded between 1995 and 2010 in Fiji and initially 294 of these cases were found to meet the criteria for CRS. Patient folders were then consulted to verify the diagnosis and classification. Folders were only available for 38 (13%) of these 294 cases. In 33 cases the review confirmed the recorded diagnosis and classification. In three cases the diagnosis was not changed but the classification was changed from “suspected” to “clinically confirmed” CRS. In two cases the diagnosis was changed from CRS to other congenital anomalies. Thus in total, 294 cases of CRS were included in the analysis.

CRS cases comprised more males than females and I-Taukei was the most common ethnic group followed by Fijians of Indian Ethnic descent (Table 1). 

**Figure 1:**
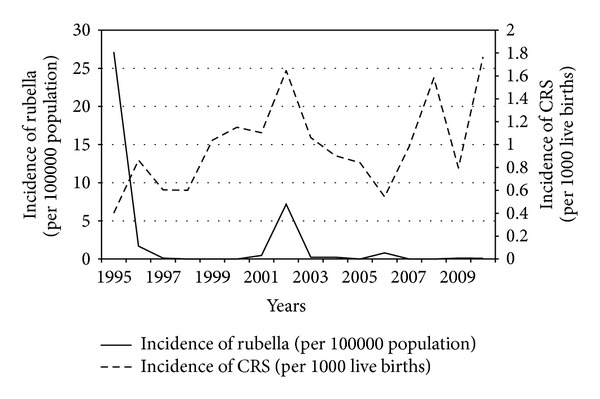
Relationship between incidence of rubella and incidence of congenital rubella syndrome in Fiji, 1995–2010.

Between 1995 and 2010, there was a significant linear increase in the incidence of CRS (odds ratio 1.045 per year, 95% CI 1.019 to 1.071, *P* < 0.001), with this incidence ranging from 0.4 cases per 1000 live births in 1995 to about 1.7 cases per 1000 live births in 2010 (Figure 1). Whilst the peak in incidence of rubella and the incidence of CRS coincided in 2002, there was no significant association between incidence of CRS and rubella (*P* = 0.3) (Figure 1).

**Figure 2:**
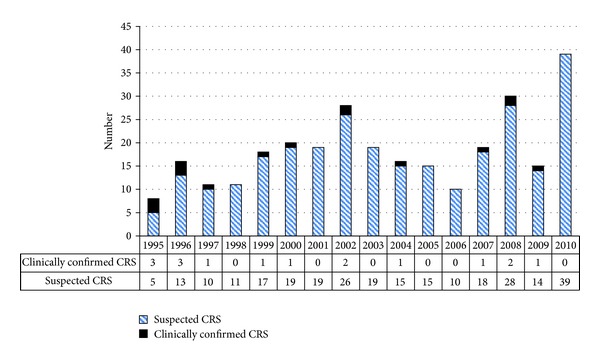
Classification of congenital rubella syndrome (CRS) by year, Fiji, 1995–2010.

The majority of CRS cases (*n* = 278, 95%) were classified as “suspected” (Figure 2). The highest annual proportions of clinically confirmed cases were registered in 1995 and 1996. Thereafter, the annual proportion of clinically confirmed cases was consistently less than 20% of total CRS cases. There were no laboratory confirmed cases. The most common presentations in babies with CRS were congenital heart disease (80%) followed by Jaundice (10%).

## 4. Discussion

This study demonstrates an increasing trend in the incidence of suspected CRS over the past 16 years. However, very few cases were clinically confirmed and there were no laboratory confirmed cases of CRS. There was no significant relationship between the incidence of CRS and the incidence of rubella. 

### 4.1. Strengths

This study is based on data for CRS recorded in all four divisions of Fiji. Hence it includes all reported cases of CRS and is therefore nationally representative. We have ensured that the data are comparable among the hospitals and with international reports by using a standard criteria, as recommended by WHO, to classify the cases.

The study adhered to STROBE guidelines [6].

### 4.2. Limitations

Validation of the diagnosis of CRS was limited in this study. There was a lack of information on clinical and laboratory aspects of the diagnosis in the registers. Only a small proportion of patient folders were available for validation purposes. Hence, we were not able to adequately assess the validity of the registers as a source of information on the diagnosis of CRS. Furthermore, not all hospitals had a congenital anomalies book and, where this did not exist, we could only obtain the diagnostic information by searching the Neonatal Intensive Care Admission Book. The information contained in this book was also limited as it did not include laboratory results or detailed clinical findings. 

### 4.3. Comparison with Previous Studies

The range in incidence of possible CRS recorded in Fiji is similar to that reported in developing countries during epidemics at about 0.6–2.2 per 1000 live births [7, 8]. This range of incidence is also similar to that of industrialized countries prior to vaccination [7, 8].

Whilst there was no significant relationship found between the incidence of rubella and incidence of CRS in Fiji, rubella outbreaks leading to increased incidences of CRS have been documented in other countries such as Panama, Oman, and Sri Lanka [8].

### 4.4. What Might Explain the Findings

The majority of the CRS cases are “suspected” cases in which the clinical finding was congenital heart disease. Improvements in diagnosis of congenital heart disease over time could be contributing to increasing numbers of cases of suspected CRS reported during this period.

The low numbers of clinically confirmed cases reported may be due to difficulties in assessment at birth particularly for features that become more apparent at a later stage of life, for example, deafness, blindness, and mental retardation.

The lack of a significant relationship between the incidence of CRS and the incidence of rubella may be caused by underreporting of rubella due to shortcomings with data recording and management at the hospitals. 

### 4.5. Implications of the Findings

The increasing incidence of suspected CRS in Fiji demonstrated through this study has implications for both clinical management and surveillance purposes. The absence of any laboratory confirmed CRS cases suggests the need to develop standard operating procedures and guidelines for confirming diagnosis through serological tests so that we are able to accurately estimate the burden of CRS in Fiji. Considering the challenges in documenting the extent of CRS in the population due to great variation in the manifestations of CRS in the first year of life and diagnostic issues related to detecting some of these features, a system would need to be established for close followup of these infants in maternal and child health clinics in order to identify features of CRS that may not be possible to assess at birth.

This study also suggests the need to monitor adequacy of rubella prevention, through the current rubella vaccination programme carried out at primary school entry. WHO recommends coverage rates to be maintained above 80% to ensure herd immunity [9]. Low coverage rates may indicate the need for serological surveys among women of child bearing age. Furthermore, assessment of the sensitivity, specificity, and predictive value of the different clinical definition of CRS may need to be carried out [7]. 

The very small proportion of patient folders obtained for validation through this study suggests the need to improve information management and record keeping at health facilities. Ultimately an improvement in surveillance of CRS would allow evaluation of our disease control efforts towards elimination of rubella and CRS. 

## 5. Conclusion

There is a rising trend in reports of suspected CRS cases in Fiji. This highlights the need to strengthen surveillance for CRS through improvements in clinical and laboratory diagnosis to confirm or exclude suspected cases. It is also important to ensure high coverage of rubella vaccination in Fiji.

## Figures and Tables

**Box 1:**
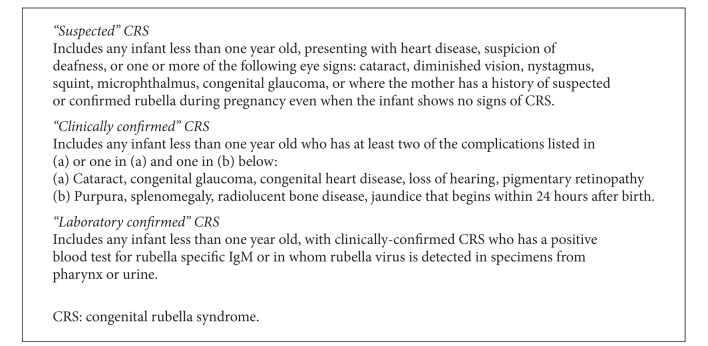
WHO diagnostic classification for CRS.

**Table 1:** Characteristics of infants with CRS in Fiji, 1995–2010.

Characteristic	Number of CRS cases* (%)
Total	294
Sex	
Males	154 (52)
Females	128 (44)
Not recorded	12 (4)

Ethnicity	
I-Taukei (indigenous population)	178 (61)
Fijians of Indian Ethnic Descent	90 (31)
Fijians of Other Ethnic Descent	14 (5)
Not recorded	12 (4)

*Number of CRS cases includes those suspected, clinically confirmed, and laboratory confirmed for CRS.

CRS: congenital rubella syndrome.
